# How the body tells the story: using the nonverbal cues of trauma between mothers and toddlers to mark a change in maternal attitudes during Clinician-Assisted Videofeedback Exposure Sessions

**DOI:** 10.3389/fpsyg.2025.1443972

**Published:** 2025-06-25

**Authors:** Suzi Tortora, Daniel Scott Schechter

**Affiliations:** ^1^Dancing Dialogue Healing and Expressive Arts, Cold Spring, NY, United States; ^2^Memorial Sloan Kettering Cancer Center, New York, NY, United States; ^3^Department of Psychiatry, Centre Hospitalier Universitaire Vaudois (CHUV), Lausanne, Switzerland; ^4^Department of Psychiatry, Université de Lausanne, Lausanne, Switzerland

**Keywords:** nonverbal analysis, video analysis, interpersonal violence, maternal PTSD, early childhood mental health

## Abstract

**Introduction:**

This study aims to examine what a form of video-based analysis of nonverbal communication: “Dyadic Attachment-based Nonverbal Communicative Expressions (DANCE)” can reveal about change in the bodies of interpersonal violence-exposed mothers who suffer from posttraumatic stress disorder. DANCE, developed by a dance-movement therapist (first author) was applied to video excerpts from a videofeedback, exposure- and mentalization-based, manualized intervention. This intervention, Clinician-Assisted Videofeedback Exposure Session(s) (CAVES),” developed by a child and adolescent psychiatrist (second author), involves the filming of mother-infant interactions. It has empirically shown a change in these mothers’ perception of their young children, as expressed verbally, but has not yet been examined for accompanying changes in behavior.

**Methods:**

Following an introduction to the theoretical premises and historical context of both DANCE and CAVES and the latter’s related 16-session manualized psychotherapy “Clinician-Assisted Videofeedback Exposure Approach Therapy (CAVEAT),” case vignettes of two mothers who underwent the CAVES with their toddlers are presented qualitatively.

**Results:**

Two case-naïve raters observed significant changes in body tone, posture, gestures and facial expression in line with previous findings of reduction of negativity of maternal attributions towards the children.

**Discussion:**

Implications for further research based on these qualitative results include consideration of (1) how the DANCE video microanalysis tool might be applied to the study of clinical progress and outcome by therapists during CAVES and related CAVEAT treatment, and (2) how more body-focused therapeutic techniques might further enhance the CAVES/CAVEAT model in future clinical applications and research.

## Introduction

Implicit to the foundation of contemporary clinical work with vulnerable families with infants and their parents is the classic quote by Selma Fraiberg, “When the mother’s own cries are heard, she can hear the cries of her child” (Fraiberg et al., 1975). While this principle of intervention has made sense in practice for nearly 50 years, the psychobiological underpinnings of why this should be so (i.e., why it works) remain a mystery. The second author (Schechter) initiated studies with colleagues first in New York and then in Switzerland to elucidate these very pertinent mechanisms. Schechter and colleagues hypothesized that mothers who have interpersonal violent trauma, including childhood maltreatment and exposure to family violence, may not only be unable to hear their infant’s cries but may also have trouble seeing the expressions on their faces as others see them. Schechter and colleagues have proposed that this is because of traumatic memory traces that interfere with perceiving the infant for whom he or she is in the present moment and context. Contemporary trauma research describes traumatic memory involving a complex dialog between the brain, mind, and body, which occurs both within and outside of the individual’s conscious awareness (Ogden et al., 2006; Porges, 2007; Porges, 2022; Rothschild, 2000; van der Kolk, 2015).

To truly “hear” and “see” both the mother and her child in the manner that Fraiberg proposes and Schechter and his colleagues investigate goes beyond analyzing spoken communication. It extends to nuances that are expressed nonverbally through body actions, gestures, and postures. Nonverbal behavior, both during and in the absence of verbal narrative, has long been noted as a powerful form of communication in the intergenerational Holocaust trauma literature that first used the term “intergenerational transmission of trauma” (Adelman, 1995; Kestenberg, 1980). Attachment research, of course, depends on the coding of nonverbal behavior in order to categorize the quality of attachment, such as in the Strange Situation Paradigm (Ainsworth et al., 1978), or variations on this procedure (Crowell and Feldman, 1988) and continuously rate both child attachment security and organization, as well as parental sensitivity that favors attachment security and organization such as the Care Index (McKinsey, 1999; Künster et al., 2010), or the Emotional Availability Scales (Biringen et al., 2000), versus frightening–frightened or otherwise “atypical maternal” behavior (Lyons-Ruth et al., 1999) that predicts insecure, disorganized attachment. And, yet, the standard coding schemes for these attachment-related aspects, while able to capture pre- to post-treatment changes with respect to attachment (Hoffman et al., 2006; Julian et al., 2018; Madigan et al., 2006; Tereno et al., 2017; Yarger et al., 2020), are often less finely tuned to microanalysis of particular moments of interaction during dyadic psychotherapy particularly with reference to the rating of each partner’s bodily tension and openness to the other, in line with concepts of “embodied mentalization” (Shai and Belsky, 2017).

This article presents a method of nonverbal analysis created by Tortora (2010, 2011, 2013) that predates embodied mentalization, and Stern’s “vitality affects” (Stern, 2010), called Dyadic Attachment-based Nonverbal Communicative Expressions (DANCE), which was initially developed to address these more subtle aspects of parent–child interactions. As will be explained in the description of the DANCE tool, what differentiates DANCE from other established coding systems that look at nonverbal behaviors, is that DANCE offers a structured approach to specifically examine the *qualitative* nonverbal elements that are at the essence of the behaviors of the individual, and the interactional patterns within a dyad. The qualitative elements create the feeling tone of the action, which can happen within or outside of the individual’s conscious awareness. DANCE helps clinicians recognize the unspoken nuances that shape both individual and shared communicative experiences. When quantified, DANCE measures moments of embodied aligned and integrated presence within the individual observed in their postures, gestures, or other actions, and/or during dyadic engagement. Used in the context of the CAVES analysis, DANCE analyzed when the quality of mom’s nonverbal behaviors demonstrated coherent moments of inner “felt” and/or mentalized reflection and personal and/or interpersonal connection that may occur out of the realm of conscious awareness to the mover and observer.

### Nonverbal video analysis approaches

The use of videofeedback to support mother–baby attachment relationships is common practice in infant mental health work for both the general public and for “at-risk” families participating in infant mental health programs (Rusconi-Serpa et al., 2009). Split screen face-to-face video analysis of parent–infant dyads to assess specific aspects of the quality of the attachment relationship has long been the standard method of analysis in the field of infant mental health (Beebe, 2003; Beebe and Steele, 2013; Harrison and Tronick, 2007; McDonough, 2004; Mesman, et al., 2008). What differs in Tortora’s method is that the focus of the analysis is the message the whole body speaks during an interactive relationship (Tortora, 2011, 2012, 2013). A full-body analysis of the parent–infant interaction reveals the implicit and embodied narrative (McGowan and Delafield-Butt, 2022), which exists as its own language that may be separate from the verbal narrative. DANCE enables the therapist to analyze the nonverbal ways the relational aspects of the dyad create both co-regulation and dysregulation and has led to innovative assessment and dance/movement therapy-based intervention strategies (Beardall et al., 2023; Tortora, 2012).

### PEM and DANCE comparison

A more recent coding system that also incorporates some aspects of full-body interaction between parents and their infants influenced by the Kestenberg Movement Profile (KMP) is Parental Embodied Mentalizing (PEM). Shai (2008, personal communication) consulted with both Tortora and Sossin during the conceptualization of PEM for her PhD studies.

Indeed, the coding of PEM draws on dance theory and movement analysis paradigms (Kestenberg Amighi et al., 1999; Laban, 1960; Tortora, 2006), so that movements of both the parent and the infant are examined closely in terms of the movement patterns that are displayed (i.e., tempo, use of space, direction of movement in space, muscle tone, and pacing of movement) and the degree to which the parent is able to infer the infant’s mental states from movement to adjust her own movement accordingly (Shai and Belsky, 2017, p. 196).

PEM is built upon the concept of parental mentalizing, which is defined as a parent’s ability to mentalize about their infant’s mental states, revealed in how they talk to and about their infants. PEM measures how a mother’s ability to envision her infant’s mental states is revealed through their nonverbal interactive behavior. This analysis has been used to predict children’s later socio-emotional functioning. “When parents fail to implicitly interpret changes in infants’ movements and rhythms as expressive mentalistic signals and fail to respond to them in an embodied manner, this compromises infants’ ability to feel—at *the embodied level*—that they are owners of their bodies and active agents capable of influencing others” (Shai and Belsky, 2011a, p. 188).

Although PEM and DANCE both analyze full-body movement, PEM differs from DANCE in several significant ways. The PEM assessment specifically focuses on dynamic communicative body movements, excluding other forms of nonverbal communication, such as gaze patterns and facial expressions, from its coding framework, and the unit of analysis is the dyad, rather than being assessed separately (Shai and Belsky, 2017). DANCE includes a wider array of nonverbal actions, including gaze and facial activity. Developed as a clinical tool, DANCE is flexible, supporting different combinations of observations in both dyadic and individual interactions, including the parent–child, therapist–client relationships, and individual participants. This study examined if the use of DANCE to analyze the mother’s full-body behavior and experience as they engage in the CAVES intervention would show alterations in maternal behavior with respect to self-regulation and reflective capacity.

PEM, more specifically, has focused on the parent’s ability to recognize and respond to an infant’s mental states through the child’s movements. It was not designed to assess parental sensitivity or attunement to all aspects of the infant’s needs (Shai and Belsky, 2011b, 2017). PEM can evaluate interactive exchanges centered on the infant’s mental states. Meanwhile, DANCE prioritizes the felt-embodied experience, viewing it as its own language. DANCE considers the nonverbal body narrative as having its own relevance in the narrative experience, occurring in tandem with mental states, but may exist outside of conscious awareness.

### Application of DANCE for CAVES

The versatile design of DANCE enables it to be used to analyze both individual and dyadic exchanges of any relational pair. For the CAVES/CAVEAT treatment, it was used to analyze the verbal and nonverbal expressions of two mothers in the research study while watching and discussing with their therapists their videotaped interactions with their toddlers. A detailed description of DANCE, including its development as it fits into the historical and recent growing research interest in movement behavior analysis with adult–infant interactions, follows this next section of the introduction, describing CAVES.

## Description of CAVES as the frame to demonstrate a coding of microanalyzed interaction to reveal possible change

Central to Schechter’s program of research has been the hypothesis that mothers with violence-related posttraumatic stress disorder (PTSD) may experience their very young child’s normative routine distress as a trigger of their memories of their aggressors (Fraiberg et al., 1975; Lieberman, 1999; Schechter et al., 2003). Schechter noted via clinical observations, a particular phenomenon in which intense displays of helplessness, frustration, rage, and terror by very young children, who by nature have limited developmental capacity to regulate their emotions, may remind mothers who have been victims of violence of either their violent perpetrators’ behavioral dyscontrol and/or the victim mother’s own fear and helplessness (Schechter et al., 2014). His hypothesis that the young child can trigger her mother’s PTSD symptoms has been supported by clinical and neuroimaging studies (Schechter et al., 2017; Schechter et al., 2015a, 2015b).

Schechter has further hypothesized that such a perceived interpersonal threat by the very young child in distress can shift a mother’s primary preoccupation with that young child’s needs to that of her own individual survival as marked by the possible fear behaviors of fight, flight, or freezing (Porges, 2007; Schechter et al., 2012). The latter significantly increases the risk of gross misinterpretation of her child’s emotional communication, both verbally and nonverbally, and this has been associated with the child’s internal dysregulation of emotion and arousal (Suardi et al., 2020). To respond to the research question of whether or not a regulated and mutually regulating, mentalizing psychotherapist can help a mother thereby to focus jointly on her child’s emotional communication, mother–child dyads participated in a simple three-session intervention, two evaluation sessions and one CAVES.

The hypothesis, which was supported as shown below, was that by the end of the three sessions of the parent–child evaluation, including CAVES, mothers would be less likely to describe their child as a threat or as a reminder of a negatively viewed, traumatizing figure in their past, thus being less affected by what Fraiberg et al. (1975) described as the maternal transference to the baby as it is made conscious.

### PTSD and negative maternal attributions

These descriptions or maternal attributions are single adjectives or short phrases that describe the child’s personality and that clue the clinician into the mother’s mental representations of her child that comprise the maternal transference to her child. Maternal representations are highly associated with how a mother will behave with her child (Zeanah et al., 2000). Whether quantitative analyses of maternal attribution quality or qualitative analyses of maternal narrative, the data analyses remain in the verbal realm. A mother’s perception of her child may be negatively skewed by the experience of interpersonal violence and subsequent triggers of post-traumatic stress (Lieberman, 1999; Lieberman et al., 2006). Schechter and colleagues’ clinical observations supported this hypothesis (Coates et al., 2003; Schechter, 2003), namely, the majority of PTSD-afflicted mothers tended to label their very young child as one of the three greatest stressors in their lives rather than as sources of joy and have distorted, negative, and poorly integrated maternal mental representations of the child (Schechter et al., 2006; Schechter et al., 2005).

### More on the CAVES

The CAVES consists of the mother and therapist trying to focus jointly—using videofeedback, on the memory of the previously filmed parent–child interaction session and examining what was most memorable, most enjoyable, and most stressful. Thereafter, the therapist, who has studied the video of the interaction session, first shows what he/she has found to be the most mutually enjoyable and reciprocal moment during free-play. This excerpt is used to build an alliance with the mother, to highlight and support her capacity for mentalization, her understanding of her child’s development, and her sensitive responsiveness during interactions. A standard series of questions is asked routinely and repeated during the CAVES by the psychotherapist, as well as a repeat of what attributions the mother would apply to describe her child’s personality. Once this first portion of the CAVES is completed, the therapist “exposes” the mother to a stressful moment, generally the most stressful for mothers, the mother–child separation. Questions and attributions are again repeated. The whole sequence is again repeated two more times, with videofeedback of the moment of reunion and then of exposure to novelty or another moment producing a negative affect on the child.

Studies of the three session intervention, including the CAVES, in support of our hypotheses, have shown a significant decrease in negativity and age-inappropriateness of the descriptive words and phrases (i.e., attributions) used to describe child personality traits, as stated in two different samples (Schechter et al., 2006; Schechter et al., 2015b). One articulate mother stated that what was mutative in this brief intervention was “seeing the expression on his face.” This mother changed her mind about what she had initially attributed to her son, namely an angry, controlling way of being as he had tried to prevent her from leaving the room—this was stated just prior to joint attention to the videofeedback of the separation film excerpt in the CAVES. She, after the CAVES, realized, “He was feeling scared… oh my God, he thought I wasn’t coming back.” During the episode, she recalled feeling stressed and unable to think. And so, it became clearer how her own sense of self-regulation had been affected by the actions and separation anxiety of her 3-year-old son, who, in fact, regularly had such severe separation anxiety that he was not able to attend daycare, let alone leave his mother alone for a few minutes to go to the bathroom.

Change in maternal perception until the present had only been measured through coding of the quality of verbal attributions and narrative. In 2018, the authors, a child and adolescent psychiatrist and clinical neuroscientist (D. S. Schechter) and a dance/movement therapist and certified Laban/Bartenieff nonverbal movement analyst (S. Tortora) discussed analyzing the mother–child interactive behavior from the mother–child evaluation session, and the maternal nonverbal behavior from the videotaped CAVES, before and after videofeedback exposure in the presence of the therapist (Tortora and Schechter, 2018).

## Aims of this study and demonstration of concept to explore behavioral change via DANCE

To explore change in nonverbal behavior pre- versus post-CAVES, the authors discussed Dr. Tortora coding the videos using her analysis system, DANCE, to detect information about the nonverbal dynamics within the mother–child attachment relationship with this vulnerable group of mothers and their toddlers. The authors’ objective was to find out what a microanalysis of the mother and child’s nonverbal actions could reveal related to the mother’s own dysregulated emotion and arousal in response to stressors, this while engaging with their young child during routine, [albeit] stressful moments. The authors also aimed to characterize how maternal behavior and self-regulatory strategies might change in line with less negative and age-inappropriate attributions toward the child, as shown in previous studies and case reports (Schechter et al., 2015a; Schechter et al., 2014).

This article presents the specific nonverbal qualities of the mother within the mother–child dyad and mother–therapist dynamic, discovered through this analysis that may provide insights into the mechanisms supporting the CAVES intervention. One of the goals of using Tortora’s DANCE tool to analyze the embodied narrative experiences of the mothers and their children with the CAVES project was to bring awareness of these potentially disruptive patterns that may be felt more than verbally articulated.

## Methods: analysis of CAVES videos using DANCE

In the present study, the two parent–child dyad videos drawn from the Geneva Early Childhood Stress Project were both analyzed using DANCE.

### Important background to the CAVES regarding methodology

The CAVES was part of the Geneva Early Childhood Stress Project from the very start, and mothers gave consent for two evaluation sessions, an MRI scanning session and CAVES on the initial consent form. The Geneva Early Childhood Stress Project was a longitudinal study examining predictors of child endophenotypic differences between children of mothers with interpersonal violence-related PTSD (vs without) whose toddlers would go on during school-age and/or pre-pubertal age to develop externalizing/aggressive versus internalizing/anxio-depressive symptoms versus children who show neither. The study specifically looked at markers of maternal self-dysregulation of emotion at three levels: psychological, physiological, and neural activity as predictors of decreased maternal sensitive caregiving behavior and, in turn, child behavioral outcomes. Additionally, the CAVES was done to see if maternal attributions of the child’s personality could become less negative and age-inappropriate following this intervention. Parent–child interaction data on video that were analyzed in this study were from the early childhood phase (child ages 12–42 months, mean age 24 months), which was completed in 2014. The protocol consisted of a screening session for mothers with a child 12–42 months of age. The index child had to have lived with his/her mother for the majority of the child’s life and be physically and mentally able to participate in the study tasks. Mothers were similarly required to speak fluent French or English, to be physically and mentally able to participate and also to be without active substance abuse or psychotic disorder. During the screening visit, mothers completed a number of paper measures, including those for the socio-demographic context. Those who qualified then returned for one mother-only visit, followed by a mother–child visit 2 weeks later, followed by an MRI scan within 2 weeks after the mother–child visit, and then the CAVES involving the mother alone 2–4 weeks later.

### Outcome measure used: DANCE—nonverbal analysis system

The DANCE observation tool developed by Tortora combines the principles of infant and early childhood mental health, dance/movement therapy, and the Laban Bartenieff movement analysis system (LBMS) (Bartenieff and Lewis, 1980) to provide a systematic way to analyze the nonverbal interactional dynamics of a dyad (Tortora, 2010, 2011, 2013). DANCE facilitates the clinician in understanding the unspoken subtleties involved in the experiences of both individual and dyadic communications. A more detailed description of DANCE and its application to code the CAVES is included in the methods section below.

### Movement metaphors: how our bodies speak

DANCE is based on the principle that our bodies hold our experiences, and the personal qualitative nuances of our body actions speak of our experiences (Tortora, 2004). These qualitative nuances are complimentary to Stern’s concept of dynamic forms of vitality. As Stern (2010, p. 11) states:

Dynamic forms of vitality are part of episodic memories and give life to the narratives we create about our lives. Accordingly, dynamic forms of vitality provide another path for psychotherapy to access non-conscious past experience, including memories, dissociated experiences, phenomenological experience, past experience known implicitly and never verbalized, and in particular “implicit relational knowing” (how we implicitly know how “to be with” a specific other)…Forms of vitality are part of all past experience… dynamic aspects are an important element of opening the past.”

How past experiences that are held in the body may be influencing the mothers’ behaviors and reactions to their toddlers is one underlying focus of CAVES. As Stern states, paying attention to the dynamic forms of vitality is a path to this past. The LBMS term *movement metaphor* analyzes the specific qualitative elements in the behaviors and can identify how these bodily held memories may influence the mover’s implicit relational knowing (Tortora, 2006, p. 77). Movement metaphors are movement sequences or postures that consistently recur in a mover’s movement signature, revealing aspects of the mover’s inner and outer experience. What is significant for the CAVES analysis, is that these actions are often a distillation of previous life experiences, especially traumatic ones, that when analyzed further, may depict the mothers’ defensive actions that are dysregulating to those mothers, and that negatively impact their reading of the of their child’s intentions and actions.

The qualitative elements of movement, meaning not just what the action is but rather how the movements are performed, provide these insights into past experiences. Again, making a parallel to Stern’s vitality forms and the LBMS system decoding movement qualities, Stern (2010, p. 127) states, “The traces of vitality forms that were experienced in the past are carried in memory. They are connected with the other aspects of remembered experience. When the vitality forms of the experience can be evoked, a whole experience can tumble out.” When analyzed, the comprehensive nonverbal patterns of movement not only elucidate how the actions are executed but also unveil potential embodied and metaphoric meanings expressed from the felt experience of feeling and moving in one’s body. The term felt experience was first discussed by Gendlin in his humanistic–experiential focusing psychotherapy six-part method (Moore, 2016, p. 188). In focusing, the client pays close attention to reactions, subtle sensations, and emotions that are not noticed in cognitive analysis alone.

### Embodiment and embodied reflective functioning as defined in dance/movement therapy

The felt experience in focusing relates to the *embodied experience* in dance/movement therapy. Dance/movement therapy regards embodiment as being rooted in the mover’s somatic narrative, which frames the psychological understanding of consciousness, unconsciousness, metaphor, and corporeality (Tortora and Keren, 2023). Embodiment in dance/movement therapy is described as a “bottom-up” approach, where sensorimotor, somatic, movement, and interoceptive sensations precede and inform thoughts and are the foundation for higher cognitive processing (Pass, 2021). A dynamic and fluid relationship exists between action, movement, creativity, emotion, and cognition, which is integral to increasing awareness and understanding of the body–mind–emotion continuum and is integral to healing (Eberhard-Kaechele, 2012; Koch and Fischman, 2011). Building from these concepts, the term embodied experience places emphasis on how one’s cognition and perception are linked to one’s physical presence and sensorial involvement with one’s surroundings. Embodiment is a state of fully engaged perception of one’s body, mind, and emotional states (Tortora and Keren, 2023).

For this study, Tortora created the term *embodied reflective functioning* to describe these instances in which the mother experiences an “*aha*” moment—an epiphany, as it were, of mentalized insight about her child or herself. This realization is typically accompanied by a spontaneous shift toward more integrated actions, reflected in her full-body posture or gestures. These shifts may or may not be in the mother’s conscious awareness and may be fleeting. Nevertheless, they are significant because they signify nonverbal, yet coherent moments of potential strength, inner reflection, personal understanding, and interpersonal connection. When brought to conscious attention during an intervention, the mother typically learns about her bodily communications, and she can build upon these nonverbal actions to create positive interactions with her child.

In this article, the authors tested the hypothesis, through comparative case reports, that analyzing CAVE videos of two mothers and their toddlers using the DANCE framework would reveal a potential relationship between moments of the mother’s reflective functioning and more fully embodied nonverbal engagement in her behavior (Tortora and Schechter, 2018).

### DANCE: a detailed description

**Figure 1:**
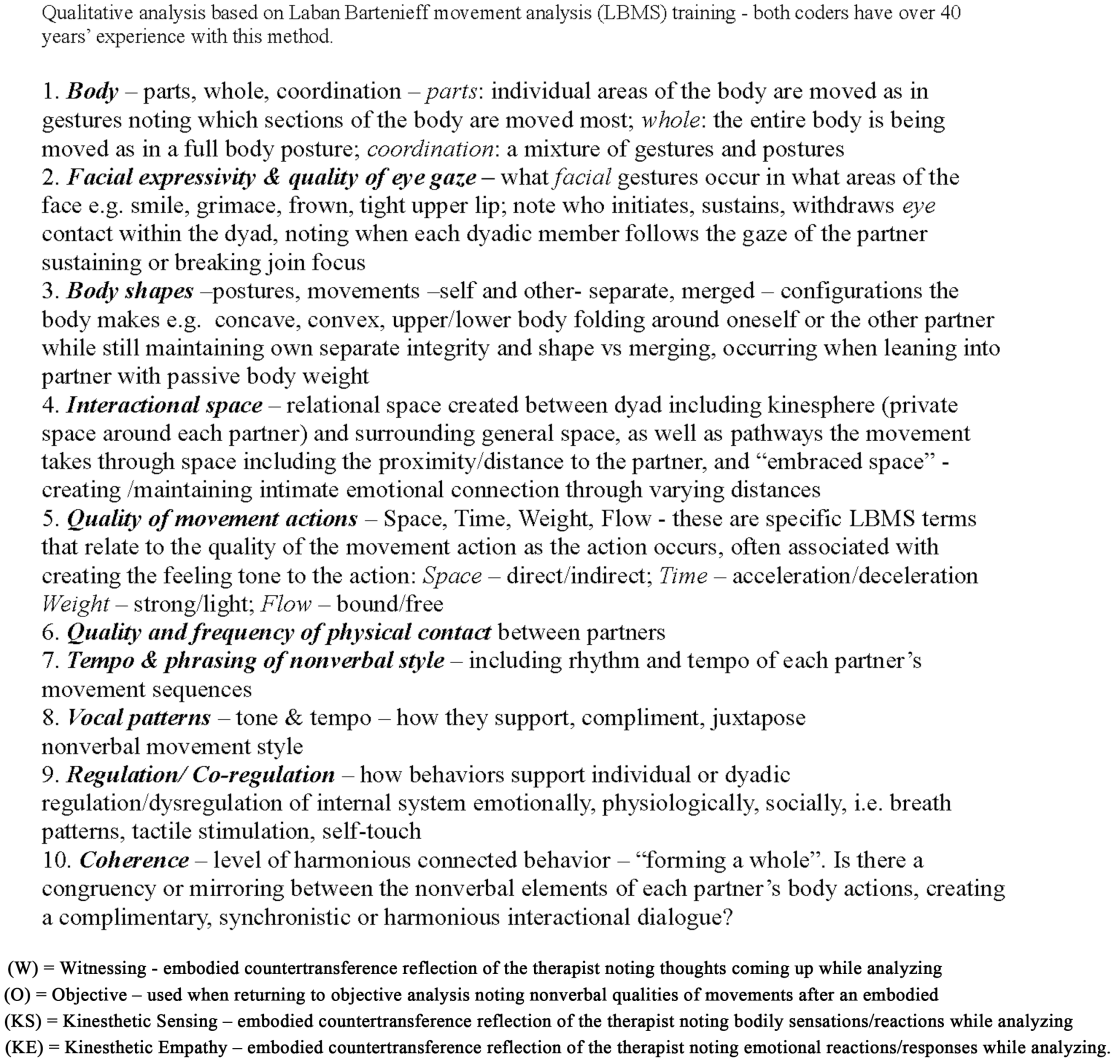
Explanation of qualitative nonverbal elements of DANCE categories: Adapted from Tortora (2011).

Going beyond verbal dialog, DANCE provides a richer, more nuanced understanding of the nonverbal tone and textural aspects of the relationship, which can easily be overlooked when concentrating solely on verbal dialog.^1^ Our nonverbal actions create a form of communication that, when looked at within the context of interactions, can provide a window into the underlying dynamics of the relationship. The 10 categories of movement analysis in DANCE provide a way to capture how the whole-body interactive exchange between two individuals contributes to that dynamic. These 10 qualitative categories analyzed in DANCE focus on the body; facial expressivity and the quality of eye gaze; specific qualities of the movement actions, including the speed, tension, flow, strength, and lightness of the actions; spatial interactional; physical contact; phrasing of the actions; vocal patterns; regulation and co-regulation; and a sense of coherence or lack of coherence within the relationship. Figure 1 provides an explanation of the elements in each category. These categories provide intricate descriptive information about the qualitative elements of the mover’s expressive actions in relationship to both their verbalizations and their interactions with the environment and those individuals they are relating to. Such details include how the movement was performed, what parts of the body were used, the rhythm and phrasing of these actions, and the context in which these qualities appeared. In its clinical use, the information gleaned from the analysis offers details about the specific nonverbal qualities of each dyadic member’s movement signature, enabling the clinician to customize the intervention to attune to each individual’s nonverbal movement style.

### Application of DANCE to CAVES video data

This coding tool was applied to the analysis of parent–child play activity and then to the videofeedback of that play activity (i.e., mom watching the play video and speaking to the therapist about her reactions, and the therapist speaking to the mom with the baby in the room). The video analysis method is an adaptation of Tortora’s (2006, p. 383) “procedure for analyzing videotaped sessions” to align with one of the overarching goals of using DANCE in the CAVES project—to invite a discussion about the narratives revealed, as well as those less apparent, through nonverbal observation, with implications for treatment, including the utilization of nonverbal analysis and videofeedback to develop intervention strategies that uncover the mother’s trauma narrative and foster a more insightful story, ultimately enhancing mother–child interactions.

A second experienced dance therapist, who was trained to be reliable in the DANCE system, coded the videos. The inter-rater reliability (IRR) was calculated using Jamovi (the jamovi project (2024). *jamovi* (Version 2.5) [Computer Software]. Retrieved from https://www.jamovi.org) on the coding of 10 out of 40 interactions coded by the two raters. The IRR was very good, with an intraclass correlation coefficient of 0.84, which translates to 91% concordance following the DANCE video analysis procedure without any discussion of the first reviewer’s (Tortora) results. The second rater, who was naïve to information about the subjects in the video data, was only told that the purpose of the current analysis was to examine these videos using DANCE as a “proof of concept” on a clinical sample of mothers and toddlers. She was told that the aim of this analysis was to demonstrate the value of assessing nonverbal behavior alongside verbal behavior, fostering a discussion about the narratives that can be uncovered—and those that might remain less apparent—through nonverbal observation. Once the second coder completed her full analysis and drew her conclusions, she and Tortora shared their qualitative data results. Their results showed that on the study videos, IRR reached 95%, with both highlighting the same timestamped sections of video for each mother and focusing on the DANCE categories: body, facial expressivity and quality of eye gaze, body shapes, interactional space, and quality of movement. The results described in this article portray their combined analysis.

### DANCE video analysis procedure

The CAVES videos were analyzed by initially watching the 3- to 5-min clips provided at least one time in real time with the sound off, timestamping sections of approximately 3–10 s in length for microanalysis. In accordance with the LBMS method, these sections were selected because they demonstrated qualitative shifts in the mother’s actions. In LBMS, qualitative elements of an action sequence are noted when there is a change in the movement quality within the sequence (Bartenieff and Lewis, 1980; Laban, 1976; Lamb and Watson, 1979).

**Figure 2:**
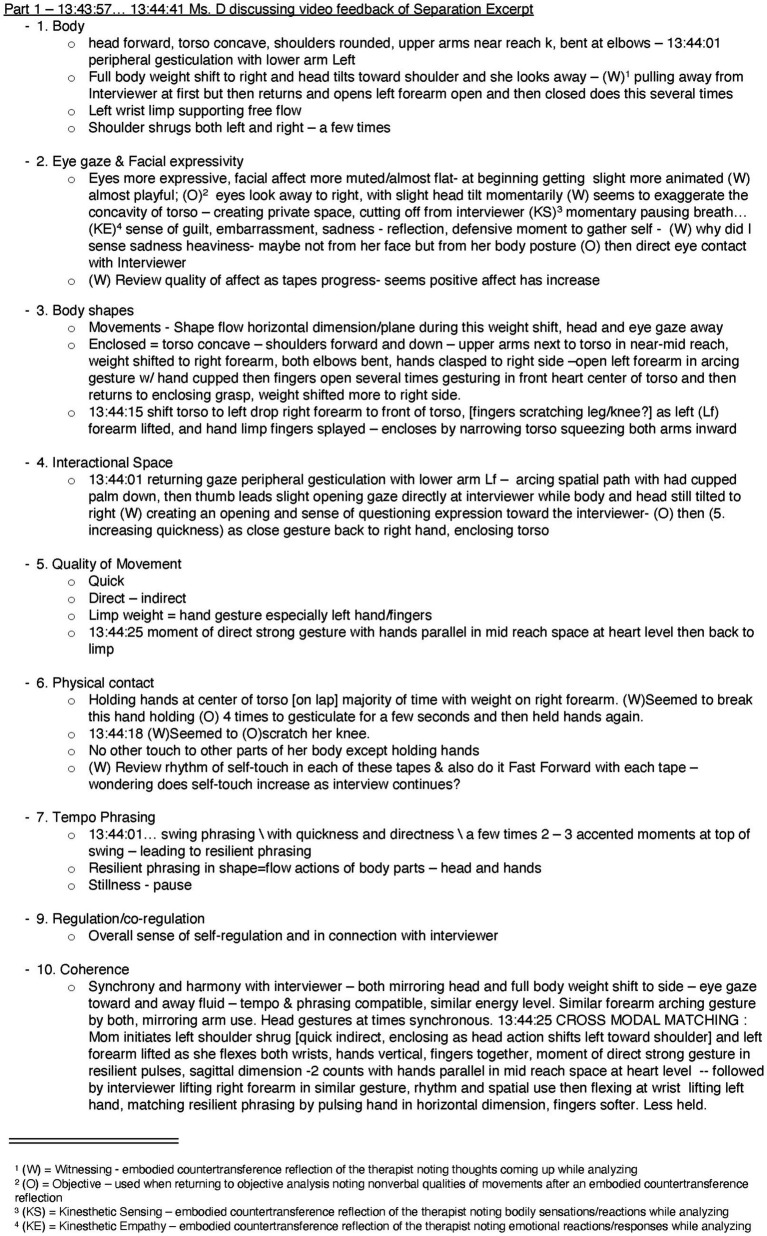
Sample DANCE note sheet: DANCE notes for CAVES Ms D.

Microanalysis of these excerpts was then also conducted with the sound off, viewing them in real time, slow motion, and fast motion approximately 4–15 times to identify the specific qualitative elements most prominent in the first eight DANCE categories. Movements are complex, and the qualitative dynamic elements are constantly in flux (Studd and Cox, 2020). Therefore, not all the DANCE categories were included in every behavior analyzed (i.e., the mover may prioritize gesturing with specific body parts over moving and shaping their body as a whole). In the final stage of the analysis, after identifying the details of the first eight categories, this information was used to evaluate the last two categories: regulation/co-regulation and coherence. Following this process, the video was reviewed again with the sound on, alongside the transcribed English dialog, to compare insights gathered from the nonverbal and verbal interactions. Figure 2 provides a sample note sheet consisting of outlined notes with the most prominent qualities noted in each category.

## Results

The authors tested the hypothesis that DANCE would reveal postural, self-regulatory and mutual regulatory differences in the mother with respect to herself, to the film of herself and her baby and to the therapist during the videofeedback portion of the CAVES. The DANCE qualitative data analysis by two trained reviewers showed a clear relationship between moments marked in the CAVES analysis as strong reflective functioning verbally, with more integrated changes in specific qualities of the mother’s nonverbal movement pattern, interpreted as positive moments of embodied knowing between the mother and the therapist. For example, at one point in the CAVES session, a mother (Ms. D, Clinical Vignette 2) exhibited a softening of muscular tension, a brightening and increase in facial expressivity, and adjustments to her posture that reduced its defensive appearance—such as relaxing her shoulders from a tense, elevated position, or transitioning from a concave stance to a more open posture, broadening her shoulders, sitting taller, and smiling. This more integrated shift in the mother’s nonverbal qualities was associated with times of increased reflective capacity and positive affect in the mother while speaking to the therapist. This was especially apparent when the content of the mother’s reflection included thoughtful and affirmative discoveries about themselves, their child, or their dyadic relationship. It was as if the mental awareness and the nonverbal experience refreshed the mother, enabling her to be more present in the moment and bringing greater emotional and physical presence for herself and her child. These moments were coded as moments of *embodied reflective functioning*, occurring when the qualities of the mother’s nonverbal/body actions support these moments of clear reflective responsiveness.

The microanalysis revealed both a congruent and lack of congruent coordination between the mother’s verbalizations and actions while speaking to the therapist when watching the mother–toddler play videos. “A lack of congruence can become apparent in two ways: through a verbal-nonverbal discrepancy or through discrepancies within the individual bodily communication in different modalities, such as smiling but with extreme body tension” (Tortora, 2011, p. 244).

For example, while watching the parent–child play video and speaking to the therapist, another mother’s laughter (Ms. A, Clinical Vignette 1) was juxtaposed with biting her lower lip in a sustained manner, abruptly picking at her nose, and swiping the back of her hand across her nose with a quick, aggressive gesture. These gestures—biting, picking, swiping performed with these nonverbal qualities—sustained, abrupt, quick, and aggressive—conflicted with our typical associations of laughter, which evokes joyful and playful actions. Similarly, a mismatch between Ms. D’s verbal and nonverbal statements was observed when she laughingly stated, “She’s scared,” while commenting on her daughter’s behavior on the video when presented with a motorized dinosaur toy. She said this with a light, brightening facial expression and subtle bounce in her whole body as she quickly lifted her head up while lengthening her torso and rocking back for a moment. The subtle bounce, light, and bright qualities, while lengthening her body, did not match the scary statement. She added an even more complex nonverbal nuance to this incongruency when she followed this statement, immediately creating an enclosed body shape by clasping her hands together and extending her arms as she bent her body over her legs and paused for 3 s, while scratching her lower leg with her right hand using a quick light, indirect action. Next, she elongated her posture, unfolding her body while pressing her hands together and bringing them to her torso, just below her lower ribs. Repeatedly analyzing this 5-s clip using microanalysis, Tortora interpreted Ms. D.’s incongruent and increasingly enclosing self-touch actions through her own embodied transferential reactions^2^ as conveying an image of self-protection and self-soothing.

## Clinical vignette 1: Ms. A and Flavio, age 14 months

### Background: clinical vignette of Ms. A and Flavio^3^

Ms. A and her son Flavio were first seen by the Geneva University Hospitals consult-liaison team when Flavio was admitted to the pediatric ward for non-organic failure-to-thrive at age 6 months. He was treated, discharged, but returned, as his weight had not been maintained. When by 9–10 months of age, it became clear that Flavio had a severe feeding disorder in the context of an attachment disturbance; an infant–parent psychotherapy was initiated with three to four sessions per week—including a dyadic focus plus individual work with both parents and infant—on the pediatrics inpatient unit. Little progress was observed both in the feeding problem and the attachment disturbance, as Ms. A did not seem to be able to link the exchange in one session to the next. Ms. A initially minimized if not denied a history of trauma in terms of maltreatment, excessive corporal punishment, exposure to domestic violence, saying that “no working-class parents’ kids” in her country of origin in Southern Europe had an easy childhood—parents could not waste time with their kids or each other because they worked hard and were strict. She would give no further details at that initial time.

Ms. A frequently mentioned new setbacks with Flavio, maintaining a negative view and ongoing frustration with him. Additionally, Flavio’s father withdrew from participation in sessions amidst mounting conflict with Flavio’s mother and the couple separated several months into the treatment as Flavio approached 12 months. Ms. A and Flavio joined the research study when Flavio reached 13 months and participated over the next 6 weeks.

### Maternal representations before the CAVES at 13 months based on the working model of the child interview responses

While Ms. A described Flavio as being affectionate, intelligent and adorable, she also described him as having a “strong personality” as being willful… and stubborn…like his dad. She said, “When it’s that one thing (that he wants), it has to be that one thing and no other!”

The comparison of this toddler to adult men that are big, strong, and to be feared emerges further when the following question was asked:

T: Whom does Flavio remind you of?

Ms. A. Well… my father because my father hit my mother a lot but even more… That makes me think of my boyfriend and at the same time, my father. Because my father is in fact also someone with a strong personality, imposing, and my boyfriend the same, the father of Flavio therefore reminds me of these two personalities because Flavio is a strong kid. It makes me think a little bit of both indeed. He is very…everyone tells me that also in fact from his face. He looks like both men….”

## Ms. A: CAVES—videofeedback of separation excerpt

Ms. A. left the room at the sound of the knock on the door and did not say anything to Flavio. He became very distressed and cried in front of the door, holding a toy cup. She continued to wait silently just on the other side of the door, holding the stopwatch and waiting 3 min before re-entering. What was striking about Ms. A.’s initial response was that she was absolutely certain that Flavio was angry with her that she left, and was trying to impose his will that she stay for him rather than let her go to the toilet. But after further intervention, she was able to see that he was sad and frightened that she had left and did not, given his developmental age, understand that she would come back. She recalled herself as a child in similar situations and was then able to empathize more with his experience.

Ms. A’s narrative had begun with a less sensitive view of her son Flavio and ended on a more empathic note, during which she was able to confront her memory of feeling frustrated, helpless and of crying as a child and adolescent. After the CAVES, mother described Flavio as intelligent, adorable …has a strong personality and active. However, an important change was noted: She replaced willful and stubborn with wary, showing Flavio to be vulnerable like her. Ms. A completed the CAVES, stating that she had not imagined that her reactions to her child were the same as those she had seen in the video. She also did not feel that they were affected by her earlier experience. She thus felt deeply moved by the CAVES and began to feel closer to Flavio.

## RESULTS: Ms. A and Flavio DANCE analysis of maternal movement

The most significant qualitative elements of Ms. A’s behaviors included full-body posturing, how and when she spontaneously shifted her body weight, and her specific self-touch gestures. These nonverbal qualities, at times, evoked a sense of containment and defensive self-protection. These postures were most prominent in response to what Ms. A had perceived as aggressive acts directed at her by Flavio, when discussing that she often experienced Flavio’s responses as frightening or startling, or when describing her own feeling of being frightened. At other times, her own actions reduced self-aggression.

A sense of containment, enclosure and self-protection appeared to be created, according to the observers, by the concavity of Ms. A’s upper body. This was suggested by Ms. A’s shoulders rolled forward and held, lifting both her shoulders in this posture and holding them for an extended time, or intermittently lifting and enclosing her left shoulder, creating a narrowing of the space between her shoulder and her head.

A startle reaction appearing self-protective in response to aggression was evoked when these postural elements were coupled with abrupt weight shifts of her whole torso to the side or backward in space. These actions especially became apparent when notating the tempo and phrasing quality using the DANCE method. At times, abrupt, explosive or impactive phrases were punctuated by sustained stillness, full-bodyweight shifts or self-touch actions.

A sense of self-protection was apparent while watching the video of Flavio crying at the door when left alone during the separation–reunion. Ms. A initially appeared to show a pleasant affect, displaying a slight smile and brightened eyes. This seemed to be a mismatch in affect relationship to Flavio’s crying. However, as she continued to observe Flavio’s distress, her self-touch gesture, a quick, light, long stroke with her left hand down her right arm, followed by a neutral affect shift facially, appeared self-soothing.

At other times, her self-touch actions, though clearly self-regulatory in nature, had an aggressive quality that, when analyzed, appeared to be unconsciously directed at herself. These actions were apparent when talking with the therapist while watching her videotaped interactions with Flavio in several vignettes. This is illustrated below in the depiction of Ms. A observing Flavio cry when left alone during the mother–child separation and then proceeding to enter and pick him up, as reflected in the DANCE coding notes.

Ms. A bites her left pinky finger/nail with a sustained strong rhythmic action and then shifts to biting her left thumb finger/nail in the same manner, while holding the rest of her body very still, with a fixed gaze on the screen. While discussing his feelings about being left alone, stating that he was angry, Ms. A bites her lower lip momentarily pressing her lips together in a strong direct manner, followed by averting her gaze momentarily and then abruptly tilting her head to an angle leaning right, lifting her eyebrows up and down quickly, and looking at the therapist from the corner of her right eye. She resumes her lip biting and pressing, looking away as the therapist asks her what she was feeling at that moment. She lifts her head up nodding, almost imperceivably as she looks directly at the therapist and states, “That’s the part that makes me most nervous.” When asked again to reflect on how Flavio felt when she returned during the separation-reunion, Ms. A first scratches her right nostril with her right hand in a quick abrupt manner and then drops it, stating “I would say a sense of relief… relief because I finally came back and took him in my arms.” This is followed by a deeper breath and softer facial expressivity, as she looks directly at the therapist.

Next, while watching herself sitting with Flavio as a novel “scary” mechanical dinosaur toy walks towards them, she uses her left hand to reach across her face to the same right side of her nose and scratches the inner nostril nose with quick short strokes manner with her pointer finger and thumb pressing against her right cheek for several seconds, with a small tight-lipped smile while holding the rest of her body very still.

A sense of self-protection was again exemplified during a game Ms. A and her toddler played (during her first Interaction Guidance psychotherapy session following the CAVES session), which involved putting their heads at each end of a long open-ended tube to watch a ball that they were taking turns rolling down the tube. When Flavio’s unskilled action rolled the ball through the tube more quickly than she expected, Ms. A suddenly and spontaneously jerked her left shoulder up in a subtle manner, creating an enclosed narrowing toward her head, as she shifted herself away and to the side of the tube stating, “Ugh, you threw it hard this time.”

The self-protective aspect of this restrained action appeared again, confirming the interpretation that Ms. A experienced Flavio’s behavior as aggression directed at her, when she described her startle reaction during this tube-play, while with her therapists in the Interaction Guidance session with Flavio. This session occurred with her two therapists following the CAVES session. As illustrated in the following description from the DANCE coding notes, both verbal discussion and nonverbal expression supported her reflection on the event and her reaction, seemingly easing her discomfort. As she recounted this incident, she sat on the floor with her right leg bent, knee facing the ceiling, and her left arm extended at her side, hand propping her up:

“… Well there…yeah it’s… he shot the first one gently and the second went faster.” Ms. A states as she extends her right leg out, opening her stance while rocking forward towards the therapist. The therapist asks “…what was going on in your mind just then” Ms. A shares, “Well, I remained in a state of shock, I guess actually, I did not understand why he had shot such a fast-ball at me…there was a normal one and then that one! Why he shot it at me so strongly!”. As Ms. A states this she slightly leaning more forward towards the therapist, holding this position for a moment before rocking her upper torso back in a larger action, creating a stronger upright posture.

Analyzing these actions more deeply, Tortora wondered if Ms. A’s initial startled reaction when Flavio rolled the ball quickly might have been a movement metaphor. Interpreting her protective defensive nonverbal stance as a movement metaphor makes this gesture more comprehensible, as evidenced by the verbal and nonverbal description below from the DANCE coding notes—a moment which occurred later in the session, when Ms. A shared her reactions about other moments with Flavio and his father that had surprised her:

Ms. A sits on the floor, leaning forward cross-legged with her shoulders hunched forward. While saying, “…at that moment, there it was more that… I did not at all expect it, in fact,” her nonverbal gestures naturally convey her surprise as her whole body shifts back, holding this posture momentarily, with her shoulders tensely lifted. She then enlarges her body stance, taking up more space, extending her right leg diagonally out, leaning further forward towards the therapists. Continuing the conversation, “But it is true that there [are] some moments when I am frightened by certain things, indeed yes…” Ms. A maintains this posture, at times pausing and then shifting back as she gestures in front of her body. Her actions are interspersed with smaller forward and backward rocking actions and symmetrical postural shifts in a more upright stance.

“But it is true that that does happen that, well, sometimes, when he wants to for example give me a hug, I get… rattled.” Again, matching her words with her actions, Ms. A, this time purposely leans backwards with more force rather than a startling reaction, demonstrating her experience of being rattled with her full body action.

Maintaining this backward shifted posture, by placing her left hand on the floor to her side, she leans further backward for emphasis stating. “But it is true that there are moments when his own gestures…make me scared, sometimes… I say to myself oh my, I am such an idiot that… well he wanted to give me a hug and me… I, like an idiot put my nose in the air and turn away… What will he think… it is the same with my boyfriend… he wants to hug me, and I said to myself, well there you have it I reacted badly once again… but afterwards, I know that it wasn’t deliberately, it’s a reflex.” Throughout this discussion, each slight shift forward precedes a further backward shift of Ms. A’s upper torso, purposefully executed with more emphasis to demonstrate her startle reaction.

As Ms. A. continued to share her feelings, the spontaneous abruptness of her startle reaction subsided, transforming into a purposeful full-body action. Observing this subtle rocking motion evoked a feeling of self-regulation for Tortora. An example of embodied reflective functioning occurred as Ms. A’s startle became a full-body experience, taking up more space, and the tension in her body appeared to settle down, matching her growing awareness and reflection. In fact, Ms. A literally and figuratively occupied more space, appearing to take in the support she was receiving through the therapists’ verbal and nonverbal communication. The therapists also attuned their bodies to her through their stillness and subtle gestures. This feeling of being supported during periods of heightened reflective capacity was observed while speaking to the therapist. As Ms. A made a positive reflective association, her posture shifted into a fuller and more physically balanced position, and her defensive startle reactions subsided and were replaced by purposeful movements on her part that matched her verbal descriptions.

In summary, Ms. A’s behavior became more open, available and deliberate, showing more agency during the Interaction Guidance session that followed that of the CAVES, which the therapists had integrated into their session with her. This important change in her behavior occurred only one time Ms. A had integrated her understanding both through her corporal and mental experience, as noted in her verbal and embodied reflective functioning. Through this process, she seemed to understand that her expectation of Flavio’s aggression was a misreading of his emotional cues and was rather based on traumatic memories with adults from her past when she was a child.

## Clinical vignette 2: Ms. D and Marta, age 15 months

### Background: clinical vignette of Ms. D and Marta^4^

Ms. D is a Swiss single 27-year-old mother of two children from different relationships: a 6-year-old son and a 15-month-old daughter. As a child, she herself suffered exposure to severe interpersonal violence that began when her mother was pregnant with her. She then ended up with violent partners. The father of her youngest child, Marta, a West African man, was particularly violent toward Ms. D, beginning after Marta’s birth. The couple separated when Marta was 12 months old. Ms. D. stayed with Marta’s father as long as she did—his violence toward her having started early in, if not just prior to the pregnancy, she said, because she rationalized that he came from a different cultural background and had not yet learned that women can be independent, trustworthy and career-oriented. She had hoped he would “change” until she feared that the violence would lead to harm to their daughter, who was increasingly agitating her father with increased demands for surveillance as she became more mobile, to the point where he struck out at Ms. D while she was holding a crying Marta in her arms.

Ms. D, as a result, developed complex PTSD and was haunted by fears that she would cross paths with her violent ex-boyfriend. She participated in the Geneva Early Childhood Stress Project with Marta. Ms. D found it difficult that Marta seemed so attached (“too attached”), while at the same time, Ms. D feared leaving Marta in daycare. She was anxious that something bad might happen to her—that someone might maltreat her. She feared being negligent.

When the research clinician met her for the CAVES, Ms. D, having participated in the maternal clinical interview and mother–child evaluation visits, was already familiar with the premises, but it was the first time she had met the CAVES therapist (Ms. Rusconi Serpa). During the interview preceding the CAVES, when responding to the *Working Model of the Child Interview* and its question about what Ms. D found most difficult to manage in her relationship with Marta, Ms. D replied, “I cannot stand it when babies cry. It means putting up in fact with being…with being helpless.” The therapist was struck by this insight, given what the therapist perceived as both Ms. D’s and her daughter’s separation anxiety. During the CAVES, after the first questions and video excerpts of a positive reciprocal interaction, the time came for the separation excerpt.

## Ms. D: CAVES—videofeedback of separation excerpt

When Ms. D heard the tapping sound indicating that she must leave the room, she stood up abruptly, speaking very quickly to Marta. Saying pleadingly: “I’m going out again, I’m leaving, I’ll be back, okay? I’ll be right back, okay? I’ll be right back.” As she left, she said “I’m coming Marta, I’m coming.” She quickly left the room. Marta stood motionless, holding a cube in her hand, and burst into intense, painful tears. Marta hardly moved during the 3 min of separation.

The therapist stopped the film and asked the following:

T: So what’s going on?
M: She’s crying, being a drama queen (chuckles).
T: You think she’s being a drama queen?
M: She’s calling, in fact she’s calling me, she’s crying to call me …
T: And that’s theatrics, do you think?
M: No, not really theatrics, but I know that when she makes those eyes, it’s because. she’s looking to get something (eyes that reminded Ms. D of Marta’s violent father).
T: Yes, she’s looking to get something. But why did you say she’s being a drama queen?
M: I do not know. She cries, in fact, she cries on purpose.

## Results: Ms. D. and Marta: DANCE analysis of maternal movement

The nonverbal qualities that appeared most salient in the analysis were Ms. D’s postural stance, self-touch, eye gaze and her use of embraced space. “Embraced space” refers to how dyadic partners sustain their emotional connection over time using varying spatial distances (Tortora, 2015b, p. 269). She appeared to be maintaining a sense of connection with her daughter Marta, and the therapist, while putting up clear boundaries. Ms. D often sat with her head forward with a slight shift to the right and her torso concave, created by rounding her shoulders forward with elbows bent, holding her hands, clasped on her lap close to her abdomen. This posture was coded as Ms. D being in a private self-contained space around herself, from which she appeared to look out and connect with others.

Tortora was taken by her own empathic reaction to Ms. D’s actions. From an interpretive perspective, Tortora questioned, why she was experiencing such sadness and heaviness while analyzing this video? Was Tortora picking up on a sense of guilt, embarrassment, sadness? Or might she have been catching a reflective or defensive moment when Ms. D was gathering herself? It did not seem to be coming from her facial expression, but rather from Ms. D’s whole body posture. These nonverbal qualities were interpreted as Ms. D’s ability, throughout the CAVES intervention, to reflect and connect to the therapist while preserving her own self-protection through self-soothing actions, creating an enclosed private, personal space around herself. The DANCE coding notes below exemplify this observation, the context of which is as follows: The therapist asked Ms. D to reflect on what she was thinking after watching the video of her pretend-play when her daughter Marta gestured for her to drink from a baby bottle and speak on the phone. In this video, Ms. D’s posture was again coded with shoulders rolled forward, creating a concave chest; however, the qualitative nonverbal aspects of Ms. D’s posture exuded a sense of vulnerability rather than defensiveness. This was attributed to the softer, less tense, and more passive weightiness of Ms. D’s shoulders and chest area (in contrast to the defensive stance of Ms. A, characterized by her stronger and tenser shoulder and chest movements). Ms. D’s vulnerable stance was created through the relationship and movement of her neck, chin, head, and arms as described below:

Ms. D. sits with her chest concave, lengthening her neck forward, by jutting her chin out while extending her arms with slightly bent elbows, symmetrically on her legs. She intermittently tilts her head to the side when looking towards and away from the therapist blinking her eye, while engaging in a variety of self-touch actions. As she crosses her right arm over her torso, she pauses, gently placing her hand on her left shoulder and softly rubbing it for twelve milliseconds. This gesture evokes a visual impression of her arm enfolding her heart like a protective scarf. Next, she clasps her hands on her lap, creating a pronounced circular enclosure of her torso by sporadically lifting and gliding her left elbow up, out and back to her torso. Ms. D continues to actively gesture in this manner at times lifting her hands, separating them with a flick of her wrists in mid-air, followed by rubbing her hands and clasping her fingers tightly on her lap, as she discusses how she feels now, while watching the video. In contemplation at one particular moment, she pauses again, drawing her elbows close to her sides, averting her gaze momentarily. Rocking side to side with a soft audible “hmmm”, she suddenly states, “That I’m a little ridiculous!” as she simultaneously looks directly back at the therapist with a bright smile and a sparkle in her eyes. In synchrony, she shifts her full body weight slightly towards her center while elongating her torso upwards and backwards. Lifting her head up and giggling, gazing straight at the therapist, with a large playful smile.

This action was Ms. D.’s most pronounced mobilization through space during this conversation, creating a nonverbal “Ah ha” moment of epiphany, in contrast to her more prominent stillness. The latter was accompanied by slight, fluid weight-shifting [described as a shape-flow quality in this nonverbal analysis system]. During this conversation, her gestures also took up more space, freely moving within the protective area within reach of her torso. This gave the impression that something had been freed for her with this epiphany, and her conversation became more reflective as the therapist provided empathic verbal support.

These gestures contrasted with her still-body posturing style seen at other times during the conversation. For example, while watching the video of their reunion after the separation during the interaction sequence, Ms. D. held her whole body in the completely concave shape described above, quite still with moments of slight tension as exhibited by furrowed brows, squinted eyes, and momentary grimaces in reaction to watching herself attempt to wipe Marta’s nose, this despite Marta’s obvious aversion to this gesture. The scene Ms. D. was watching on the video was noted as follows:

Ms. D enters the room and Marta is crying vigorously. Sitting on the floor, arms distance away, Ms. D extends her arms reaching out to Marta. Lifting her up, she places Marta within her crossed legs. Marta stands within the open space Mom’s legs create. resting her hands on Ms. D’s shoulders, with their bodies slightly against each other. Ms. D wraps her right arm around Marta’s torso, while caressing Marta’s head with her left hand, hugging her more closely, pressing their bodies together.

Still crying Marta slightly pulls her head back away from Ms. D’s face. Ms. D uses this moment to gather a tissue in her left hand, while using her right hand to attempt to wipe Marta’s nose. Immediately Marta arches her back, struggling to turn her head away from Ms. D. She returns her left hand to Marta’s head, restricting Marta’s actions, as Marta turns her head away to the right. With a sweeping action, Ms. D wipes Marta’s nose more firmly. Next, she adjusts Marta’s tense body, placing her across her lap, as Marta, holding her own body stiffer, does not mold to Ms. D’s body, giving the impression of remaining separate from Ms. D.

Ms. D’s nonverbal revelation, coherence and connection with the therapist was thus again evident, immediately after the video was turned off. She continued to hold her whole body still, while momentarily glancing at the therapist and then away with a slight grimace and head-shake, as the therapist stated “There…” and questions, “So what is going on there?”

Contemplating this question Ms. D states “Yeah, and so…actually, I noticed that I blow her nose while I could have done that later “. She says this, while, chuckling, dropping her shoulders, leaning back while lengthening her whole body up, and pulling her elbows in as if hugging her ribcage, while smiling directly at the therapist.

This was coded as Ms. D’s body expression matching her realization and regret that she had been wiping Marta’s nose rather than comforting her. As they continued this discussion, with the therapist encouraging her by pointing out, “Very interesting! You see how useful it is to watch things,” Ms. D, became reflective, as expressed both verbally and nonverbally in the following DANCE coding notes:

Ms. D’s hand gestures increase as she glides them separated across her legs, bringing them to chest height, as she flexes her wrists and flip flops her hands symmetrically in rhythm to her statement, “Yeah, because I tell to myself, she’s still crying, and I know she doesn’t like when I blow her nose, and thus I …. her, sorry for the word, but I bug her even more, and I tell myself “I could have done that later.” Grasping her right thumb with her left fingers and pausing, she presses her clasped hands against her chest. Next pausing, with her wrists flexed while holding her clasped fingers in mid-space she creates an embraced container around her body – again evoking the image of wrapping a scarf around her body - by holding her bent elbows at her ribcage. Increasing her pressure on this grasp, Ms. D follows this action with a firm tug as she pulls her thumb away from her own grasp, clasping her hands together again as she momentarily drops them on her lap; and immediately returns them to her lower rib cage, pressing them while clasping and slightly twisting her wrists. Holding them in this manner she gazes at the therapist, with a small close-mouthed smile and her head tilted towards her right shoulder.

As they continued to discuss Ms. D’s action, her reflections became even more insightful, as she stated, “I thought I was doing the right thing. Actually, I see that, I calm her down by talking to her but the gesture, it does not fit with that actually.” Ms. D’s self-regulating actions were again apparent when she stated “well, if she understands me, I would have said “sorry, sorry” well…” and the therapist said, “You wanted to apologize right?” As portrayed below in the DANCE coding notes:

Ms. D slightly tilts her upper body towards the therapist, facing her and folding her arms across her chest, hugging herself as she glides her hands up and down her upper arms, with a flat resigned smile as she looks directly at the therapist with a softness in her eyes, [seemly asking for forgiveness] followed by a giggle, and an opened smile quickly looking away and then back again as the therapist states “We can understand.” Ms. D’s head and neck are now more aligned and the concavity in her chest is less evident.

## Discussion

This article tested the authors’ hypothesis that a nonverbal analysis using DANCE would reveal differences in bodily, self-regulatory, and mutual regulatory behaviors in the mothers in a brief video-feedback-exposure intervention CAVES across three contexts: her interactions with herself, her responses to the video of herself and her toddler, and her engagement with the therapist during the video-feedback portion of the CAVES. The DANCE data, indeed, demonstrated an association between moments identified in the CAVES analysis as instances of strong verbal reflective functioning and positive moments of *nonverbal embodied knowing* between the mother and therapist. These moments created a constructive and supportive connection between the mother and the therapist during treatment. During these single interviews, the nonverbal analysis revealed mothers’ behaviors changes, assuming less defensive, victimizing and protective postures, during specific supportive moments of the intervention. During moments of increased parental reflective functioning, the mothers presented a similar shift nonverbally that could be interpreted as a spontaneous release or opening on a felt experiential level. What was important about these embodied reflective moments was that with the therapist’s support, the mothers were able to consider a new, more age-appropriate understanding of their child’s reactions, rather than misinterpreting these behaviors as manipulations or aggressive “acting out.”

Using DANCE with dyads exposed to traumas related to interpersonal violence and maltreatment created a systematic, detailed way to decipher elements within their actions that may have been contributing to the interactional dynamics within the dyad. This was the first time such a nuanced analysis of the qualitative elements of nonverbal behaviors had been used in this clinical population.

DANCE provided insight into the nature of the mother–child attachment relationship and the role of self and co-regulation within this group of violence-exposed mothers and their toddlers. The presentation of this movement analysis opened a discussion of the story that can be gleaned and not as well-gleaned otherwise.

The findings highlight important implications for treatment, emphasizing the use of nonverbal analysis and videofeedback to design intervention strategies that uncover the mother’s trauma narrative and foster a more insightful and cohesive story, ultimately improving mother–child interactions. Both mothers, suffering from complex IPV-related PTSD, displayed negative and age-inappropriate attributions toward their toddlers’ personalities. Ms. A, in the first example, saw her son as controlling and threatening, such that she felt she must retain a hypervigilant and defensive physical posture. Ms. D, in the second example, saw her daughter as a manipulative, “drama queen” who was not truly distressed upon separation, but rather exaggerating—if not feigning, her distress with an expression or “look” in her eyes that was reminiscent of Ms. D’s violent ex-partner, Marta’s father. Both mothers felt initially helpless and menaced or victimized and through the work during the CAVES and subsequently, in the case of Ms. A came to see, rather, the helplessness and anxiety in their children, and their misreading of child-cues due to confusion with violent perpetrators from the mothers’ past.

### Defensive use of cultural differences to minimize the severity of interpersonal violence

In line with the theme of the current special issue focusing on cultural dimensions of parent–infant mental health, the authors have noted how the mothers in both clinical vignettes defensively attempted to minimize or deny the severity of maltreatment and domestic violence in their histories in their initial narrative by using the not uncommon defense that “all parents” in the case of Ms. A’s native Iberian culture verbally assault and hit their children. In the case of Ms. D, all men in her partner’s North African Islamic culture treat women badly, feeling that women cannot be trusted (Nadan et al., 2015; Renteln, 2005). The mothers used this defense despite the fact that both the CAVES and the mothers’ nonverbal behavior before and after the CAVES, as revealed by the DANCE analysis, showed the effects of what could only be described as physical abuse and domestic violence histories in their respective childhoods and adulthoods on themselves and on their relationship with their child.

More specifically, Ms. A told pediatric hospital staff that she had no more a rough childhood than anyone from her social milieu in her country of origin—and remained tough and even “imposing” in her posture as a suggestion that she was not someone who could be described as having been helpless or victimized. The specific questions and persistence of questioning about life events, including maltreatment and domestic violence exposure, enabled Ms. A to speak more openly about her trauma history and the CAVES, followed by continued treatment in Interaction Guidance, allowed her to reveal that she perceived her young son’s approaches for affection as potential attacks for which, as shown by DANCE, she physically braced herself.

Ms. D, rather than using cultural differences from the therapist(s) as a defensive posture, intellectualized that her violent partner’s being from a North African Islamic culture meant that he had predetermined ideas about women’s roles and innate risk for infidelity. She imagined that this was why he mistreated her violently, as if to say it was not his fault as an individual, that his culture was rather to blame. Increasingly dependent on him, in part with roots in her own European background of violence exposure, she imagined and hoped Marta’s father “would change” as he became more acculturated in Switzerland. This rationalization also protected Ms. D from imagining that her daughter Marta might become either aggressive like her father or victimized like women in his native country because Marta was being raised solely by Ms. D in Switzerland.

Not to go to the other extreme of saying that violence perpetration and victimization are independent of all cultural influences, of course, the tolerance of intrafamilial violent acts, with blame assumed by the victim, may be supported by a collective that influences that already traumatized patient; even though this may place the children at greater risk for maltreatment (Ara et al., 2023; Cho et al., 2024). The belief that one should not or even cannot abandon one’s partner/spouse, even if he is violent, due to cultural and/or religious pressures is similarly upheld in some cultures and religious groups, the members of which the authors have also seen in clinical consultation (Ara et al., 2023; National Academies of Sciences, Engineering, and Medicine, Division of Behavioral and Social Sciences and Education, Health and Medicine Division, Committee on Law and Justice, Board on Children, Youth, and Families, Board on Global Health, & Forum on Global Violence Prevention, 2018; Nikbakht Nasrabadi et al., 2014).

### What nonverbal analysis via DANCE has contributed to our understanding of CAVES

Through the nonverbal analysis, the authors have presented two contrasting clinical vignettes. What is especially interesting is that through the microanalysis, what appeared at first glance to be similar maternal postures—tense forward shoulders creating a concavity in the chest—the specific qualitative elements of these postures and supporting actions created different maternal body language messages in response to their children’s behaviors. In the first of Ms. A and Flavio, Dr. Tortora observed in Ms. A’s bodily movement and expression, self-protective and self-regulatory gestures that fit with her verbal narrative that she saw her son through the lens of her traumatic memories with other men in her life; namely, her father and Flavio’s father, who she perceived both as imposing and aggressive. Her full-body postures and gestures portrayed frightened behaviors in reaction to her son. This hardened defensive posture only softened in the interactive guidance session after CAVES, analyzing the play interaction with Flavio when he rolled the ball “too hard” through the tube, triggering his mother’s startled reaction.

In the second vignette of Ms. D and Marta, Ms. D was able to show curiosity as to why she did not wait to wipe her daughter’s nose after the stressful separation. Similarly to Ms. A, Ms. D had manifested a concave, self-protective posture that opened up with the therapist’s validation of Ms. D’s observations of her and Marta’s behavior. Here, though, her postures and gestures initially portrayed more aggressive or even frightening actions toward her daughter, rather than being frightened by her child. Tortora described that during her “Ah ha” moments, Ms. D expanded her physical presence by elongating and centering her torso. She also gestured more freely, accompanied by her spontaneous, vibrant facial expressions. In both examples, the two mothers’ affect regulation dysfunction was also portrayed through the movement analysis. When able to demonstrate improved reflective mental capacities and curiosity about their child’s experience, improved and more integrated embodied self-regulation was observed as they confronted their own responses to their child’s emotional communication, while accompanied by the therapist who was also mutually regulating.

This study suggests that applying the DANCE methodology to code nonverbal behavior before and after interventions could lend itself both to qualitative and quantitative research in the future. An example of such an application might apply to a current open trial in progress of CAVEAT with interpersonal violence-exposed mothers with complex PTSD and their children ages 1–4, a 16-session manualized psychotherapy which is based on the technique of intervention CAVES described in the present article (Schechter, 2024).

## Conclusion: putting nonverbal and verbal analyses together

In summary, what was shown in previous research articles is that CAVES contributes to change in the quality of maternal verbal attributions toward her child’s personality, namely with reduction of negative quality and age-inappropriateness of the descriptions of the child’s personality (Schechter et al., 2006; Schechter et al., 2015a). We know that these verbal attributions reflect how the mother is perceiving and thinking about the child. The present study has shown that, parallel to this verbal expression of maternal perception, the embodied experience of her feelings about her child is also portrayed in her nonverbal communication with her child (Avdi et al., 2020; Shai and Belsky, 2011b). This nonverbal communication, by extension, contributes to her actions with her child and changes with intervention, as noted by coding *via* DANCE. The interaction of mentalization and embodied experience plays an essential role in the overall experience between the child, who is developing his/her sense of self, and the mother, who is perceiving and responding to the child according to her perception (Shai and Belsky, 2017).

This study has, furthermore, highlighted the role of the body *in* mentalization, also by introducing the idea that the felt experience may actually precede or—at the very least, simultaneously inform the mentalization process. We have asserted that felt experience in the body and mind must be considered together to fully understand and support the mother’s experience. The language of the body reflects a change in the conscious and unconscious and/or dissociated realizations of the mind. The analyses we have presented here demonstrate a relationship between the nonverbal behavioral change and verbal attributions, suggesting that it is the combination of the felt experiential change happening together with the mentalizing awareness that bolsters the mother’s qualitative change in perception. In essence, body and thought inform each other (van der Kolk, 2015).

Adding these dimensions to intervention strategies can lead to more creative approaches that include whole-body and experiential activities. In dance/movement therapy, for example, the use of movement, rhythm, dance, and music activities is used to create a dancing dialog, promoting positive communication, and enhancing social engagement and self–expression, while developing both the parent’s and baby’s mutual- and self-regulatory skills (Tortora, 2015a). In conclusion, this article asserts that considering verbal attributions and nonverbal behavioral changes together provides a richer and more nuanced understanding of the mother’s experience and the mechanisms of change and potential healing of the parent–child relationship.

## Data Availability

Publicly available datasets were analyzed in this study. This data can be found as follows: The data and specifics of the methods used that support the conclusions of this article will be made available by the authors, without reservation.

## Footnotes

^1^A description of the development of DANCE can be found in Beardall et al. (2023).

^2^Staying attuned to the dance therapist’s own embodied countertransference reactions is an integral aspect of the nonverbal analysis process and is exemplified as Tortora’s reflections in this article.

^3^Summarized with permission from the more detailed case study in Schechter and Rusconi-Serpa (2014).

^4^Summarized with permission from Rusconi Serpa S, unpublished, University of Paris, University Diploma in Attachment Studies (Rusconi Serpa, 2015).
